# Image Completion in Embedded Space Using Multistage Tensor Ring Decomposition

**DOI:** 10.3389/frai.2021.687176

**Published:** 2021-08-13

**Authors:** Farnaz Sedighin, Andrzej Cichocki

**Affiliations:** ^1^ Medical Image and Signal Processing Research Center, School of Advanced Technologies in Medicine, Isfahan University of Medical Sciences, Isfahan, Iran; ^2^ Computational and Data Intensive Science and Engineering Department, Skolkovo Institute of Science and Technology (SKOLTECH), Moscow, Russia; ^3^ Systems Research Institute, Polish Academy of Science, Warsaw, Poland

**Keywords:** tensor ring decomposition, image completion, multistage strategy, tensorization, Hankelization

## Abstract

Tensor Completion is an important problem in big data processing. Usually, data acquired from different aspects of a multimodal phenomenon or different sensors are incomplete due to different reasons such as noise, low sampling rate or human mistake. In this situation, recovering the missing or uncertain elements of the incomplete dataset is an important step for efficient data processing. In this paper, a new completion approach using Tensor Ring (TR) decomposition in the embedded space has been proposed. In the proposed approach, the incomplete data tensor is first transformed into a higher order tensor using the block Hankelization method. Then the higher order tensor is completed using TR decomposition with rank incremental and multistage strategy. Simulation results show the effectiveness of the proposed approach compared to the state of the art completion algorithms, especially for very high missing ratios and noisy cases.

## 1 Introduction

Joint analysis of datasets recorded by different sensors is an effective approach for investigating a physical phenomenon. For this propose, the acquired datasets can be recorded in different matrices and jointly analyzed (Ozerov and Févotte, 2009).

By increasing the volume of the recorded data and also the number of the recorded signals, matrices are not any more efficient for analyzing and representing the datasets. For overcoming this limitation, the acquired datasets are represented in tensor formats. Tensors are higher order arrays used in different signal processing applications such as Blind Source Separation (BSS) (Cichocki et al., 2009; Bousse et al., 2016), image in-painting/completion (Liu et al., 2012; Yokota et al., 2016; Wang et al., 2017), time series analysis (Kouchaki et al., 2014; Sedighin et al., 2020) and machine learning (Rabanser et al., 2017; Sidiropoulos et al., 2017).

The acquired datasets can be incomplete or uncertain due to different reasons such as noise, outliers, hardware failure or human error (Liu et al., 2012; Yokota et al., 2016; Wang et al., 2017). Even, sometimes, the resolution of the recorded data is not sufficient due to hardware limitations. In these situations, tensor completion can be very helpful for efficient data analysis.

Tensor completion is an approach for recovering missing or uncertain elements of a data tensor using its available elements (Liu et al., 2012; Yokota et al., 2016; Wang et al., 2017). At the first look, the problem seems to be ill-posed and difficult, but indeed, it can be done using some assumptions for the original data tensor (Gandy et al., 2011; Liu et al., 2012; Bengua et al., 2017).

Generally, tensor completion approaches can be divided into two groups: low rank based approaches (Liu et al., 2012; Bengua et al., 2017) and tensor decomposition based approaches (Yokota et al., 2016, Yokota et al., 2018). However, in some completion methods such as (Yuan et al., 2018b), both of the approaches have been combined.

In low rank based completion approaches, it is assumed that the original tensor is low rank. Under this assumption, the completion is to find the missing elements of a tensor in a way that the completed tensor has low rank approximation (Signoretto et al., 2010; Gandy et al., 2011; Liu et al., 2012; Bengua et al., 2017; Yuan et al., 2018b). In contrast to matrices, there is no unique definition for the tensor rank. One of the basic definitions for the tensor rank is the Canonical Polyadic Decomposition (CPD) rank, which is the number of rank-one tensors after CP decomposition of the original tensor. Estimating the CPD rank of a tensor is difficult, therefore, other alternative definitions such as weighted summation of the ranks of different unfoldings of the data tensor have been proposed (Liu et al., 2012). Algorithms such as Simple Low Rank Tensor Completion (SiLRTC) or High accuracy Low Rank Tensor Completion (HaLRTC) (Liu et al., 2012; Bengua et al., 2017) can be considered as examples for rank minimization based completion algorithms.

In tensor decomposition based approaches, the incomplete tensor is decomposed into several lower order or smaller factor matrices and/or core tensors. These latent factors usually preserve the structural information of the original tensor. Therefore, the completed tensor can be reconstructed using the estimated latent factors. Existing different approaches for tensor decomposition, result in different decomposition based completion approaches. In (Yokota et al., 2016), a completion approach based on CPD has been proposed. In (Yokota et al., 2018), Tucker decomposition has been exploited for tensor completion.

**FIGURE 1:**
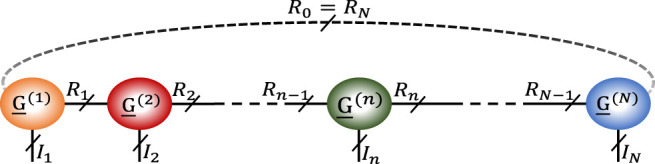
General structure for TT and TR decompositions. For the TT decomposition, $R_{0}=R_{N}=1$.

Besides the mentioned tensor decomposition based approaches, recently, new completion methods based on Tensor Train (TT) (Grasedyck et al., 2015; Ko et al., 2018; Yuan et al., 2019) and Tensor Ring (TR) (Wang et al., 2017; Yuan et al., 2018a; Yuan et al., 2018b) decompositions have also been proposed. TT decomposition is an approach for decomposing a tensor into a series of inter-connected third order core tensors (Oseledets, 2011), as (see Figure 1).

$$\underline{X}≃\ll {\underline{G}}^{\left(1\right)},{\underline{G}}^{\left(2\right)},\ldots ,{\underline{G}}^{\left(N\right)}\gg ,$$ (1)

where $\underline{X}\in \;{\mathbb{R}}^{I_{1}\times I_{2}\times \cdots \times I_{N}}$ is the *N*‐th order tensor to be decomposed and ${\underline{G}}^{\left(n\right)}\in \;{\mathbb{R}}^{R_{n-1}\times I_{n}\times R_{n}}$ is the *n*‐th core tensor. The vector [$R_{0},R_{1},\ldots ,R_{N}$] is the TT rank vector and $R_{0}=R_{N}=1$ (Oseledets, 2011). The $\left(i_{1},\ldots ,i_{N}\right)$-th element of $\underline{X}$, i.e., $x_{i_{1},\ldots ,i_{N}}$, can be estimated as

$$x_{i_{1},\ldots ,i_{N}}≃G_{i_{1}}^{\left(1\right)}G_{i_{2}}^{\left(2\right)}\cdot \cdot \cdot G_{i_{N}}^{\left(N\right)},$$ (2)

where $G_{i_{n}}^{\left(n\right)}$ is the *i*_*n*_-th lateral slice of the *n*‐th core tensor.

In TR decomposition, similar to TT decomposition, a tensor is decomposed into a chain of inter-connected third order core tensors with a notation similar to (Eq. 1), with, $R_{0}=R_{N}\geq \;1$ (Zhao et al., 2016). The elements of a tensor with TR structure can be computed as

$$x_{i_{1},\ldots ,i_{N}}≃\text{tr}\left(G_{i_{1}}^{\left(1\right)}G_{i_{2}}^{\left(2\right)}\cdot \cdot \cdot G_{i_{N}}^{\left(N\right)}\right),$$ (3)

where “tr” denotes the trace operator.

Recently, a promising approach, called Hankelization, has been developed for improving the quality of tensor completion approaches (Yokota et al., 2018; Sedighin et al., 2020). Previously, Hankelization has been used as an initial step for a time series analysis algorithm, called Singular Spectrum Analysis (SSA) (Golyandina et al., 2013; Rahmani et al., 2016; Hassani et al., 2017; Kalantari et al., 2018). Generally speaking, Hankelization is an approach for transferring a lower order dataset to a higher order one by exploiting Hankel matrix structure. In this approach, the initial data is reshaped into a tensor whose slices have Hankel or block Hankel structure. Recall that in a matrix with Hankel structure, all of the elements in each skew diagonal are the same. Hankelization provides possibility of exploiting local correlations of the elements. Due to the Hankel structure, it is expected that the resulting higher order tensor provided by the Hankelization is low rank. This low-rankness in addition to a rank incremental strategy for Tucker model has been used for tensor completion in (Yokota et al., 2018). In (Asante-Mensah et al., 2020), TR decomposition has been used for the completion of Hankelized images. Also in (Sedighin et al., 2020), a new approach for time series completion based on a two-stage Hankelization has been proposed. In this approach, a one way data, i.e., the time series, is first reformatted into a Hankel matrix, and then the resulting Hankel matrix is block Hankelized into a 6-th order tensor using block (patch) Hankelization. In block Hankelization approach, in contrast to classic Hankelization methods, blocks of elements are Hankelized instead of individual elements (Sedighin et al., 2020).

In this paper, we mainly focus on image completion. Using the block Hankelization approach of (Sedighin et al., 2020), the original incomplete tensor (image) is first transformed into a higher order 7-D tensor. Then a TR decomposition has been applied for image completion. Applying TR decomposition for the completion of Hankelized images, or better to say, in the embedded space, has been investigated in few papers such as (Asante-Mensah et al., 2020) and (Sedighin et al., 2021). Similar to many TT and TR completion approaches, determining proper ranks for completion is an important issue, so in this paper, the rank incremental strategy, developed by us in (Sedighin et al., 2021), is used for determining the TR ranks. Moreover, a multistage strategy is developed for further improving the quality of reconstructed images. Multistage strategy has been previously proposed by us in (Sedighin et al., 2020) for time series completion. To best of our knowledge, this is the first time of using multistage strategy for image completion along with adaptive TR decomposition. This is the main difference of the proposed approach with the simulation presented in (Sedighin et al., 2021). The contributions of this paper can be summarized as.

• Applying block Hankelization on each frontal slice of the incomplete image which results in a 7-th order tensor. The block (patch) Hankelization has been applied on each slice of the color image separately, which results in three 6-th order tensors. Then these 6-th order tensors have been combined with each other to produce a 7-th order tensor
• Applying TR decomposition with automatic rank incremental strategy proposed in (Sedighin et al., 2021).
• Developing a multistage strategy for improving the quality of the final reconstructed image.

Extensive simulation results confirmed the quality of the proposed algorithm in image completion, especially for high missing ratios and noisy cases.

The rest of this paper is organized as follows: Notations and preliminaries are presented in Section 2, different TT and TR completion algorithms are briefly reviewed in Section 3. Section 4 is devoted to the Block Hankelization and the proposed algorithm is presented in Section 5. Finally, results and discussion are presented in Section 6 and Section 7, respectively.

## 2 Notations and Preliminaries

Notations used in this paper are adopted from (Cichocki et al., 2016). It is assumed that vectors, matrices and tensors contain real valued elements. An *N*‐th order tensor is denoted by a bold underlined capital letter as $\underline{X}\in \;{\mathbb{R}}^{I_{1}\times I_{2}\times \cdots \times I_{N}}$, where $I_{n}$ is the number of elements in the *n*‐th mode. An $I_{1}\times I_{2}$ matrix is denoted by a bold capital letter as $X\in \;{\mathbb{R}}^{I_{1}\times I_{2}}$ and vectors are denoted by bold lower case letters as $\text{x}\in \;{\mathbb{R}}^{I_{1}}$. The $\left(i_{1},i_{2},\ldots ,i_{N}\right)$-th element of tensor $\underline{X}$ is denoted by $x_{i_{1},i_{2},\ldots ,i_{N}}$.

Matricization or unfolding of a tensor is reshaping that tensor into a matrix with the same elements. The mode-n matricization of a tensor is defined as $X_{\left(n\right)}\in \;{\mathbb{R}}^{I_{n}\times I_{1}\cdot \cdot \cdot I_{n-1}I_{n+1}\cdot \cdot \cdot I_{N}}$ in which $X_{\left(n\right)}\left(i_{n},\bar{i_{1}\cdots i_{n-1}i_{n+1}\cdots i_{N}}\right)=x_{i_{1},i_{2},\ldots ,i_{N}}$. Mode-{n} canonical matricization of a tensor is also defined as $X_{<n>}\in \;{\mathbb{R}}^{I_{1}I_{2}\ldots I_{n}\times I_{n+1}\ldots I_{N}}$ in which $X_{<n>}\left(\bar{i_{1}\cdot \cdot \cdot i_{n}},\bar{i_{n+1}\cdot \cdot \cdot i_{N}}\right)=x_{i_{1},i_{2},\ldots ,i_{N}}$. Furthermore, $X_{\left[n\right]}\in \;{\mathbb{R}}^{I_{n}\times I_{n+1}\cdots I_{N}I_{1}\cdots I_{n-1}}$ is an unfolding of a tensor in a way that $X_{\left[n\right]}\left(i_{n},\bar{i_{n+1}\cdot \cdot \cdot i_{N}i_{1}\cdot \cdot \cdot i_{n-1}}\right)=x_{i_{1},i_{2},\ldots ,i_{N}}$.

Hadamard (component wise) product of two tensors is denoted by ⊛. Mode-*n* product of a tensor $\underline{A}$ and a matrix **B** is denoted by $\underline{A}\times_{n}B$. vec(X) and rank(X) are the vectorization and rank of a matrix, respectively.

## 3 Tensor Train and Tensor Ring Based Completion Algorithms

Tensor Train decomposition has been exploited in many completion algorithms. In (Yuan et al., 2019), two completion algorithms, TT Weighted OPTimization (TT-WOPT) and TT Stochastic Gradient Descent (TT-SGD) have been proposed. The two algorithms are based on the minimization of the following weighted cost function using a gradient descent method:

$${\left\|\underline{\Omega }⊛\left(\underline{X}-\hat{\underline{X}}\left(\theta \right)\right)\right\|}_{F}^{2},$$ (4)

where $\underline{\Omega }$ is a binary mask tensor whose elements are 1 for the observed and 0 for the missing elements, $\underline{X}$ is the observed tensor and $\hat{\underline{X}}\left(\theta \right)$ is the estimated tensor with TT structure as

$$\hat{\underline{X}}\left(\theta \right)=\ll {\underline{G}}^{\left(1\right)},\ldots ,{\underline{G}}^{\left(N\right)}\gg ,$$ (5)

and $\theta =\left({\underline{G}}^{\left(1\right)},\ldots ,{\underline{G}}^{\left(N\right)}\right)$

A TT based completion algorithm using system identification has been proposed in (Ko et al., 2018). In this approach, the indices of the observed elements are the inputs of a system and the observed values are the outputs. Alternating Least Squares (ALS) and Alternating Directions Fitting (ADF) based approaches have also been proposed in (Grasedyck et al., 2015).

TT rank minimization has been used in many of tensor completion algorithms. Indeed, these algorithms are basically categorized as the rank minimization based approaches, however, since they are using TT rank which is closely related to TT decomposition, we have briefly reviewed them in this section.

In these algorithms, the cost function is defined as (Bengua et al., 2017)

$$\overset{N-1}{\underset{n=1}{\sum }}\alpha_{n}\text{rank}\left(X_{<n>}\right),$$ (6)

where $\alpha_{n}$ is the weight of the *n*‐th term. The above cost function is a weighted summation of the ranks of the canonical unfoldings of the input tensor, called TT rank. Algorithms, such as SiLRTC-TT and TMac-TT have been developed based on the minimization of the TT rank and parallel matrix factorization (Bengua et al., 2017). Furthermore, minimization of the TT rank in addition to a sparsity assumption of the mode-n matricizations of the incomplete tensor has been proposed in (Yang et al., 2018).

TR decomposition has also been used in many completion algorithms. TR-WOPT has been proposed in (Yuan et al., 2018a) and is based on minimizing the cost function similar to (Eq. 4), where $\underline{X}\left(\theta \right)$ has a TR structure. In (Wang et al., 2017), an approach called TR-ALS (Alternating Least Squares) has also been developed for tensor completion.

TR-LRF (TR Low Rank Factors) algorithm has been proposed in (Yuan et al., 2018b). This algorithm is based on a combination of rank minimization and TR decomposition approaches, in which the rank minimization has been applied on different matricizations of the core tensors resulting from TR decomposition of the incomplete tensor.

Moreover, in (Yu et al., 2018), a completion algorithm called MTRD has been introduced based on a more balanced matricization of a tensor with TR structure. A new matricization has been defined as $X_{<n,d>}\in \;{\mathbb{R}}^{I_{a}I_{a+1}\cdot \cdot \cdot I_{n}\times I_{n+1}I_{n+2}\cdot \cdot \cdot I_{a-1}}$, where

$$X_{<n,d>}\left(\bar{i_{a}i_{a+1}\cdot \cdot \cdot i_{n}},\bar{i_{n+1}i_{n+2}\cdot \cdot \cdot i_{a-1}}\right)=x_{i_{1},i_{2},\ldots ,i_{N}},$$ (7)

and *a* is defined as

$$a=\{\begin{array}{l} n-d+1\;\;\;\;d\leq n\\ n-d+1+N\;\;\text{otherwise}. \end{array}$$ (8)

In (Yu et al., 2019), another completion approach, called TRNNM (TR Nuclear Norm Minimization), based on minimizing the nuclear norm of $X_{<n,d>}$ has been proposed. Other completion approaches based on balanced matricizations of tensors with TR structure have also been proposed in (Huang et al., 2020a,b).

## 4 Block Hankelization

**FIGURE 2:**
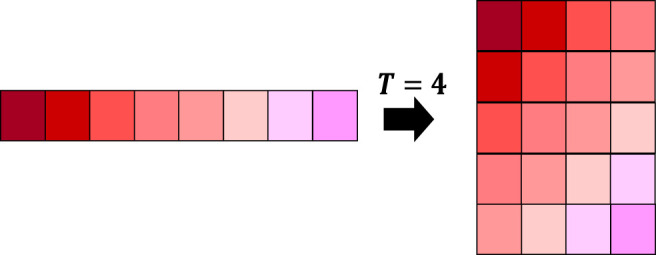
Illustration of Hankelization of a one-dimensional signal.

As mentioned earlier, Hankelization is an effective approach in signal processing for exploiting local correlations of pixels or elements. In many of time series completion or forecasting algorithms, Hankelization has been used for transforming a lower order signal into a higher order matrix or tensor (Shi et al., 2020). An illustration for Hankelizing a time series has been shown in Figure 2.

In (Yokota et al., 2018), Hankelization is performed by multiplying a special matrix, called duplication matrix, by each of the modes of the original tensor followed by a tensorization step.

**FIGURE 3:**
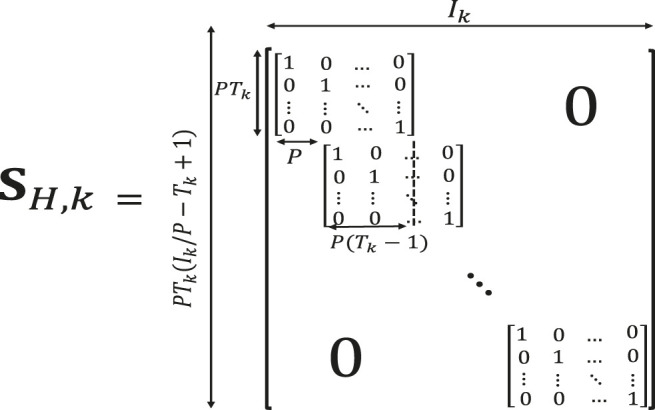
Illustration of a block duplication matrix. The lower order tensor is transformed into a higher order tensor by multiplying this matrix into its different modes following by a folding step.

Block (patch) Hankelization using blocks of elements (instead of single elements) has been proposed in (Sedighin et al., 2020). The block Hankelization is also performed by multiplying matrices, called block duplication matrices (shown in Figure 3), by different modes of the original tensor (Sedighin et al., 2020). Each block duplication matrix is of size $\begin{array}{l} S_{H,k}\in \mathbb{R}PT_{k\left(I_{k}/P-T_{k}+1\right)\times I_{k}} \end{array}$, where $S_{H,k}$ is the block duplication matrix which is multiplied by the *k*-th mode of the original tensor, *P* is the block size, $T_{k}$ is the *k*-th window size and $I_{k}$ is the size of the *k*-th mode of the original tensor which should be dividable by *P*.

**FIGURE 4:**
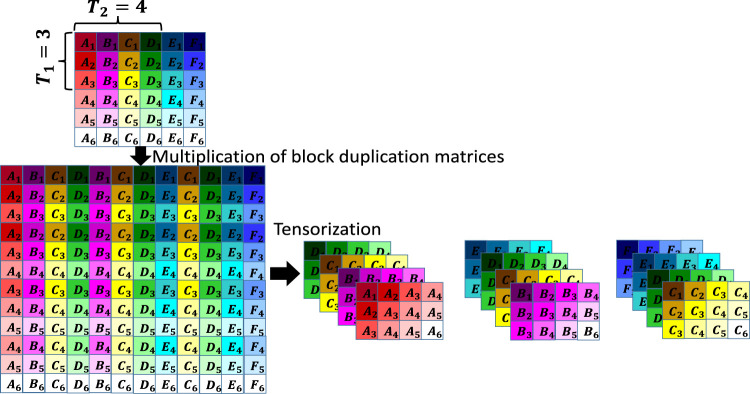
Illustration of the block Hankelization. In the first step, the initial matrix is multiplied by two block duplication matrices, and in the second step, the resulting matrix is folded into a 6-th order $P\times P\times T_{1}\times D_{1}\times T_{2}\times D_{2}$ tensor, where each colored block is a P×P matrix.

The resulting matrix after multiplication of matrix $H\in \;{\mathbb{R}}^{I_{1}\times I_{2}}$ by block duplication matrices $S_{H,1}\in \;{\mathbb{R}}^{PT_{1}\left(I_{1}/P-T_{1}+1\right)\times I_{1}}$ and $S_{H,2}^{T}\in \;{\mathbb{R}}^{I_{2}\times PT_{2}\left(I_{2}/P-T_{2}+1\right)}$ is $H_{b}=S_{H,1}{\mathrm{HS}}_{H,2}^{T}\in {\mathbb{R}}^{PT_{1}\left(I_{1}/P-T_{1}+1\right)\times PT_{2}\left(I_{2}/P-T_{2}+1\right)}$. The matrix $H_{b}$ is then folded (tensorized) into a 6-th order tensor of size $P\times P\times T_{1}\times D_{1}\times T_{2}\times D_{2}$, where P×P is the block size, $T_{1}$ and $T_{2}$ are the window sizes for the first and second modes, respectively, $D_{1}=I_{1}/P-T_{1}+1$ and $D_{2}=I_{2}/P-T_{2}+1$. Block Hankelization has been illustrated in Figure 4. Note that in this figure, each colored block is a P×P matrix [for more details please see (Sedighin et al., 2020)].

## 5 Proposed Algorithm

As mentioned earlier, the main idea of this paper is to apply TR decomposition on the Hankelized incomplete image. In many TR completion algorithms, the cost function used for completion can be written as

$$\begin{array}{c} J\left(\theta \right)={\left||\underline{\Omega }⊛\left(\underline{X}-\underline{\hat{X}}\left(\theta \right)\right)|\right|}_{F}^{2}, \end{array}$$ (9)

where $\underline{\Omega }$ is the mask tensor, whose elements are 1 for the observed and 0 for the missing elements of the input tensor, $\underline{X}$ is the incomplete input tensor, $\begin{array}{l} \underline{\hat{X}} \end{array}$ is the completed tensor with TR representation and $\theta =\left({\underline{G}}^{\left(1\right)},{\underline{G}}^{\left(2\right)},\ldots ,{\underline{G}}^{\left(N\right)}\right)$. The cost function in the embedded space, i.e., after Hankelization of the datasets, can be written as

$$\begin{array}{c} J_{H}\left(\theta \right)={\left||{\underline{\Omega }}_{H}⊛\left(\underline{H}-\underline{\hat{H}}\left(\theta \right)\right)|\right|}_{F}^{2}, \end{array}$$ (10)

where ${\underline{\Omega }}_{H}$ and $\underline{H}$ are the resulting tensors after Hankelization of $\underline{\Omega }$ and $\underline{X}$, respectively and $\hat{\underline{H}}\left(\theta \right)$ is the TR estimation of $\underline{H}$.

Similar to (Yokota et al., 2018) and by using a Majorization Minimization approach, minimization of (Eq. 10), can be achieved by minimizing the following auxiliary function

$$J_{H}\left(\theta |\theta^{k}\right)={\left|\left|{\underline{\Omega }}_{H}⊛\left(\underline{H}-\hat{\underline{H}}\left(\theta \right)\right)\right|\right|}_{F}^{2}+||\left(\underline{1}-{\underline{\Omega }}_{H}\right)⊛{\left(\hat{\underline{H}}\left(\theta^{k}\right)-\hat{\underline{H}}\left(\theta \right)\right)||}_{F}^{2},$$ (11)

where $\hat{\underline{H}}\left(\theta^{k}\right)$ is the estimated tensor in the TR format in the *k*-th iteration and $\underline{1}$ is a tensor with the size equal to ${\underline{\Omega }}_{H}$ whose all elements are 1. The cost function (Eq. 11), can be re-written as (Yokota et al., 2018; Sedighin et al., 2020)

$$J_{H}\left(\theta |\theta^{k}\right)=||\tilde{\underline{H}}-\hat{\underline{H}}{\left(\theta \right)||}_{F}^{2},$$ (12)

where $\begin{array}{l} \tilde{\underline{H}}\;={\underline{\Omega }}_{H}⊛\underline{H}+\left(\underline{1}-{\underline{\Omega }}_{H}\right)⊛\underline{\hat{H}}\left(\theta^{k}\right) \end{array}$. It can be inferred from (Eq. 12) that the minimization of (Eq. 10) is equivalent to the TR decomposition of the input tensor, where in each iteration, the observed elements are kept fixed. Considering (Eq. 12), TR estimation is done in an iterative manner, where the number of iterations is denoted by $I_{int}$. In each iteration, the TR decomposition of $\tilde{\underline{H}}$, i.e., $\hat{\underline{H}}\left(\theta \right)$ has been estimated and then the updated $\tilde{\underline{H}}$ is computed.

As mentioned in the introduction, determining the ranks for TT and TR decompositions is a challenging task. In papers such as (Wang et al., 2017; Yuan et al., 2018b), the TR ranks are determined in advance and as inputs for the algorithm. These fixed rank approaches usually fail in reconstruction of the images with high missing ratios. Also, determining proper input ranks is difficult. In papers such as (Yokota et al., 2018; Sedighin et al., 2020), the input ranks are not determined in advance, but are increased gradually during iterations. Experiments show that these adaptive rank approaches are more successful in image completion when missing ratio is high. In this paper, for controlling the TR ranks, i.e., the size of the core tensors, rank incremental approach, presented in detail in (Sedighin et al., 2021), has been exploited. In (Sedighin et al., 2021), sensitivities of the estimation error to each of the core tensors are estimated and the size of the core tensors whose sensitivities are larger than a threshold are increased. However, in contrast to (Sedighin et al., 2021), in this paper, both of the ranks of the selected core tensor are increased. The step for increasing the ranks in each iteration can be 1 or any other natural number. It is also possible not to increase the ranks in some iterations, i.e., changing the rank increasing step to 0. The latter case is mainly applicable for images with high missing ratios.

**FIGURE 5:**
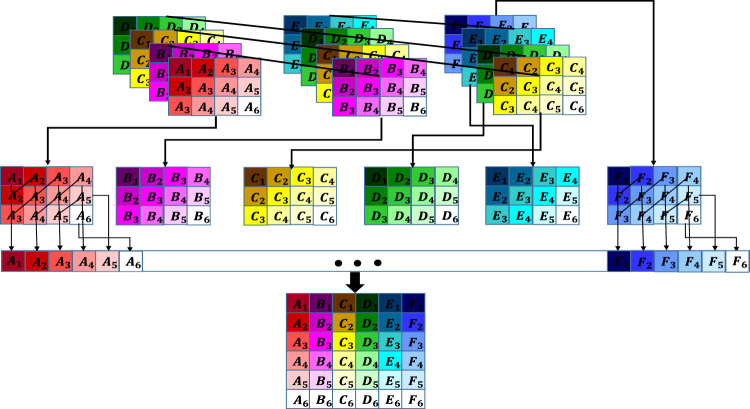
Illustration of the de-Hankelization of a block Hankelized dataset. Each colored block is a P×P matrix.

The estimated $\hat{\underline{H}}$ is then re-transformed into the original image space by de-Hankelization, which results in $\hat{\underline{X}}$. De-Hankelization is a procedure for transferring back a Hankelized dataset into its original format. De-Hankelization of a block Hankelized tensor is done by averaging the corresponding frontal slices (slices with the same color in Figure 4) and then averaging the blocks with the same color and alphabet (Sedighin et al., 2020). An illustration for de-Hankelization has been shown in Figure 5. The estimated $\hat{\underline{X}}$ is then modified as $\hat{\underline{X}}=\underline{\Omega }⊛\underline{X}+\left(\underline{1}-\underline{\Omega }\right)⊛\hat{\underline{X}}$ and smoothness, by replacing each estimated element of $\hat{\underline{X}}$ (for $\underline{\Omega }=0$) by the average of its four neighbors in the frontal slice, is applied.

The above procedure is repeated until the maximum value of the TR rank vector achieves the limit or the normalized approximation error in the embedded space i.e., $\frac{{\left||{\underline{\Omega }}_{H}⊛\left(\underline{H}-\hat{\underline{H}}\left(\theta \right)\right)|\right|}_{F}^{2}}{{\left|\left|{\underline{\Omega }}_{H}⊛\left(\underline{H}\right)\right|\right|}_{F}^{2}}$ (or the change in the normalized approximation error between two consecutive iterations) becomes less than a pre-determined error level ϵ.

**FIGURE 6:**
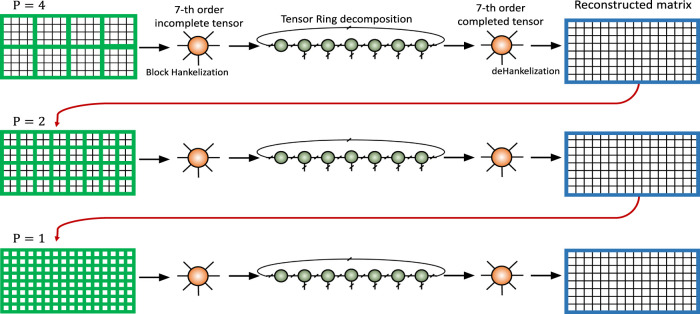
Conceptual model for the multistage strategy in the embedded space.

In this paper, we have employed a multistage strategy to further improve the quality of final results. This is the main difference of this paper with the simulation presented in (Sedighin et al., 2021), which was the result of a single stage algorithm. The main idea and the structure of the multistage strategy have been illustrated in Figure 6. In this approach, the incomplete image is block Hankelized with block size $P_{1}\times P_{1}$ and completed by the algorithm. Then the output of the algorithm is again processed as the new input of the algorithm with block size $P_{2}\times P_{2}$, where $P_{2}<P_{1}$. The initial TR rank vector for the first stage is set to all 1 vector. For the next stages, the initial values of the rank vectors are set higher than 1 but lower than the final ranks of the previous stage. The procedure can be repeated for several stages until the desired accuracy is achieved. The multistage strategy has a benefit of providing a good initialization for the algorithm and improving the quality of the final result (especially for very high missing ratios and noisy cases), in comparison to a single stage algorithm.

The pseudo code of the proposed algorithm has been listed in Algorithm 1.

**Algorithm 1:** Pseudo code of the proposed algorithm

**INPUT:** Incomplete image $\underline{X}$, mask tensor $\underline{\Omega }$, vector of block sizes $p=\left[P_{1},P_{2},\ldots ,P_{L}\right]$ where *L* is number of stages, $t=\left[T_{1},T_{2}\right]$, rank vectors $r_{1},\ldots ,r_{N}$, error level ϵ, maximum value of the rank $R_{max}$, internal iteration number $I_{int}$, the rank increasing step (inc) and the threshold for selecting the core tensors for rank incremental in each iteration (tol). **OUTPUT:** Completed image $\hat{\underline{X}}$. 1: Initialize the missing elements of $\underline{X}$ by zero. 2: **for** l=1:L **do** 3: Block Hankelize the input incomplete image ($\underline{X}$) and the mask tensor ($\underline{\Omega }$) by block size $P_{l}\times P_{l}$ and window size $t=\left[T_{1},T_{2}\right]$ which results in $\underline{H}$ and ${\underline{\Omega }}_{H}$. 4: Put $\tilde{\underline{H}}=\underline{H}$ 5: **while** $\text{max}\left(r_{l}\right)<R_{max}$ (or the normalized approximation error is higher than the error level ϵ) **do** 6: **for** $j=1:I_{int}$ **do** 7: Compute the TR decomposition of $\tilde{\underline{H}}$, i.e., $\hat{\underline{H}}$ with rank vector $r_{l}$. 8: $\tilde{\underline{H}}={\underline{\Omega }}_{H}⊛\underline{H}+\left(\underline{1}-{\underline{\Omega }}_{H}\right)⊛\hat{\underline{H}}$ 9: **end for** 10: Increase the elements of the rank vector $r_{l}$ using the approach of (Sedighin et al., 2021). 11: Compute $\hat{\underline{X}}$ by de‐Hankelizing $\tilde{\underline{H}}$ (in noisy cases by de‐Hankelizing $\hat{\underline{H}}$). 12: $\hat{\underline{X}}=\underline{\Omega }⊛\underline{X}+\left(\underline{1}-\underline{\Omega }\right)⊛\hat{\underline{X}}$. 13: Apply smoothing by replacing each estimated element (for $\underline{\Omega }=0$) by the average of its four neighbors in the frontal slice and keeping the observed elements fixed. 14: **end while** 15: Put $\underline{X}=\hat{\underline{X}}$. 16: **end for**

## 6 Results

In this section, the effectiveness of the proposed multistage approach has been investigated and compared with the state of the art algorithms. The proposed algorithm has been compared with the HaLRTC algorithm (Liu et al., 2012) which is a rank minimization based completion algorithm and also with MDT (Yokota et al., 2018), TR-ALS (Wang et al., 2017), TT-WOPT (Yuan et al., 2018a) and TR-LRF (Yuan et al., 2018b), which are tensor decomposition based approaches. We have used standard (typical) color images for our simulations.

In the first simulation, the approaches have been compared for the completion of images with high missing ratios, i.e., 90%, 95% and 99% missing pixels. The parameters regarding to each algorithm have been listed in Table 1. The results have been presented in Tables 2–4, respectively. Peak Signal to Noise Ratio (PSNR), Structural SIMilarity (SSIM) and normalized approximation error $\left(\frac{{\left|\left|\underline{T}-\hat{\underline{X}}\right|\right|}_{F}}{{\left|\left|\underline{T}\right|\right|}_{F}}\right)$, where $\underline{T}$ is the original image, have been written beneath each figure in brackets. For the proposed approach, the two stage strategy has been exploited. The block size of the first stage was set to $P_{1}=8$ and for the second stage was set to $P_{2}=4$. The window size of the both stages was t=[2,2]. Rank incremental step for 90% and 95% missing ratios were 1 for both of the stages. For 99% missing ratio, in the first stage, the ranks were increased by 1 in each 4 iterations, and for the second stage the rank increasing step was 1 in each iteration. The size of the reconstructed images in Table 2 and Table 3 are 128×128×3 and in Table 4 are 256×256×3. For the MDT approach, the window size was set to [8, 8, 1] for 128×128×3 images and [16, 16, 1] for 256×256×3 images. The initial rank vector for TR-LRF was set to [5,5,5] and for TT-WOPT has been set to [15,15,3]. In TR-ALS, the initial rank for 90% missing ratio was set to [3,3,3] and for 95% missing ratio was set to [25,25,25] (Table 1). As the results show, for high missing ratios, the proposed approach provides higher performance in comparison to the fixed rank approaches such as TT-WOPT, TR-ALS and TR-LRF. This higher performance is more significant for 99% missing ratio where TR-ALS completely fails. Indeed, the TR-ALS algorithm could not provide any output for images with 99% missing ratio. This shows the importance of adaptive rank selection for image completion. The higher performance of the algorithm is also clear in comparison with the low rank completion based algorithm such as HaLRTC. The results show that the performance of HaLRTC dramatically deteriorates when the missing ratio increases. The proposed algorithm has also slightly higher performance in comparison to the MDT approach, which is a tensor decomposition based approach with rank incremental strategy. The run time of our algorithm is larger than the other considered approaches. This is due to the multistage structure of our algorithm. Additionally, our algorithm in each stage has inner iterations for computing TR decomposition of the input and outer iterations for increasing the ranks. These two kinds of iterations are repeated for each stage. Moreover, in our approach 3-rd order images are transformed into 7-th order tensors. Analyzing this higher order tensor also increases the run time of the algorithm. These together increase the run time of our approach, however this longer run time results in the better performance of our approach.

**TABLE 1:** The initial parameters for each algorithm for the first simulation.

Missing ratio	90%	95%	99%
Image size	128×128×3	128×128×3	256×256×3
	$P_{1}=8$, $P_{2}=4$, **t** = [2,2]	$P_{1}=8$, $P_{2}=4$, **t** = [2,2]	$P_{1}=8$, $P_{2}=4$, **t** = [2,2]
Proposed	$R_{max}\leq 23$	$R_{max}\leq 23$	$R_{max}\leq 23$
Algorithm	Initial rank = [1,1,1,1,1,1,1]	Initial rank = [1,1,1,1,1,1,1]	Initial rank = [1,1,1,1,1,1,1]
MDT	Window size = [8,8,1]	Window size = [8,8,1]	Window size = [16,16,1]
TRLRF	Rank vector = [5,5,5]	Rank vector = [5,5,5]	Rank vector = [5,5,5]
TR-ALS	Rank vector = [3,3,3]	Rank vector = [25,25,25]	—
TT-WOPT	Rank vector = [15,15,3]	Rank vector = [15,15,3]	Rank vector = [15,15,3]

**TABLE 2:** Comparison of the performance of the algorithms for the reconstruction of images with 90% missing ratio. PSNR, SSIM and normalized approximation error corresponding to each image have been written in brackets.

Missed image	HaLRTC	TT-WOPT	TR-ALS	TR-LRF	MDT	Proposed
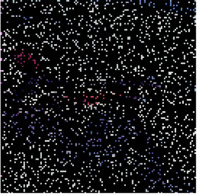	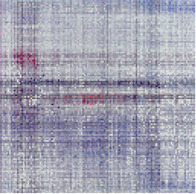	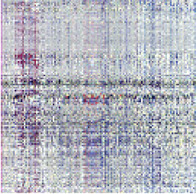	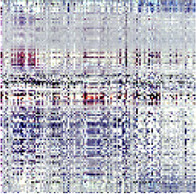	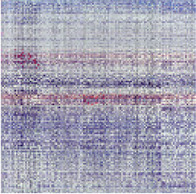	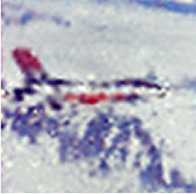	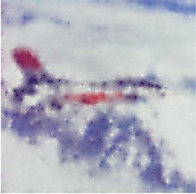
[3.2,0.01,0.94]	[16.2,0.3,0.21]	[15.1,0.2,0.23]	[8.3,0.1,0.52]	[15,0.38,0.37]	[21.5,0.73,0.11]	[**22.3,0.77,0.1**]
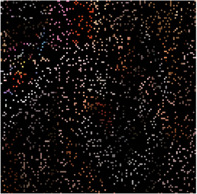	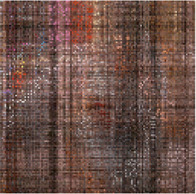	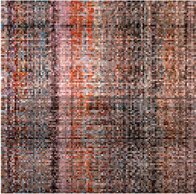	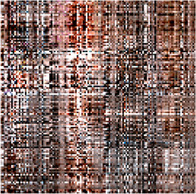	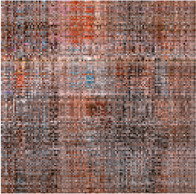	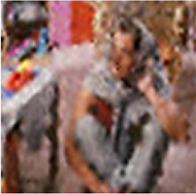	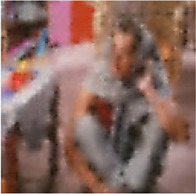
[7,0.027,0.94]	[14.4,0.44,0.4]	[13.9,0.3,0.42]	[6.4,0.4,0.98]	[15.4,0.4,0.35]	[21.8,0.8,0.17]	[**22.4,0.8,0.16**]
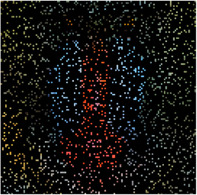	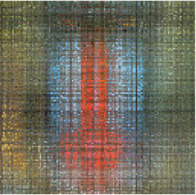	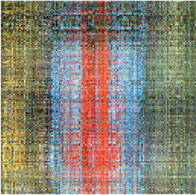	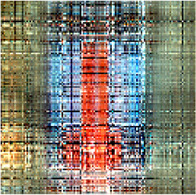	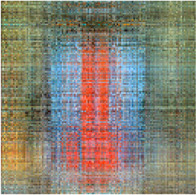	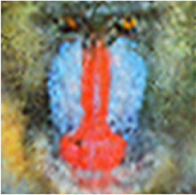	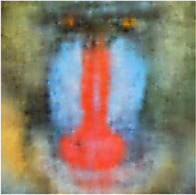
[6,0.02,0.94]	[15.3,0.44,0.3]	[15.2,0.4,0.3]	[10.2,0.3,0.57]	[17.1,0.5,0.26]	[21.2,0.73,0.16]	[**22.3,0.76,0.14**]

**TABLE 3:** Comparison of the performance of the algorithms for the reconstruction of images with 95% missing ratio. PSNR, SSIM and normalized approximation error corresponding to each image have been written in brackets.

Missed image	HaLRTC	TT-WOPT	TR-ALS	TR-LRF	MDT	Proposed
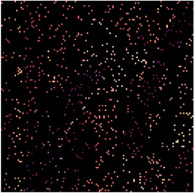	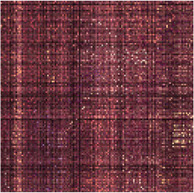	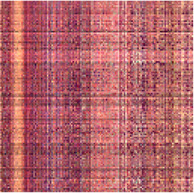	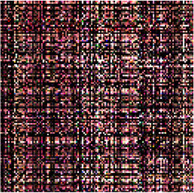	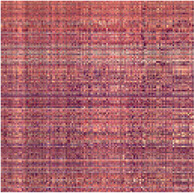	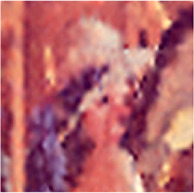	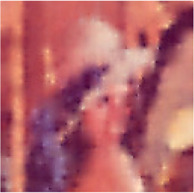
[5.4,0.01,0.97]	[10.8,0.5,0.5]	[14.6,0.6,0.3]	[4.5,0.06,1]	[14.8,0.64,0.3]	[20.96,0.89,0.16]	[**21.22,0.89,0.15**]
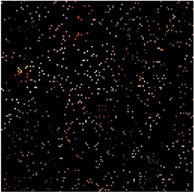	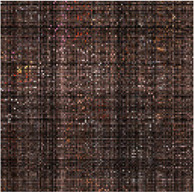	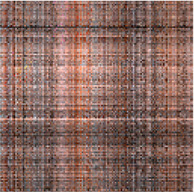	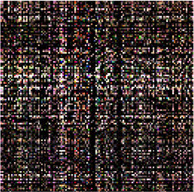	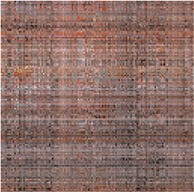	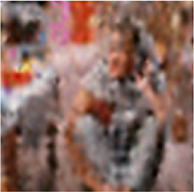	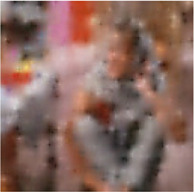
[6.7,0.01,0.97]	[10.8,0.2,0.6]	[14.1,0.3,0.4]	[5.9,0.03,1]	[14.7,0.36,0.4]	[19.8,0.73,0.21]	[**20,0.73,0.21**]
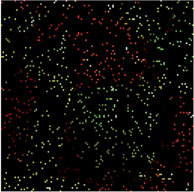	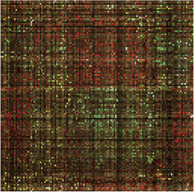	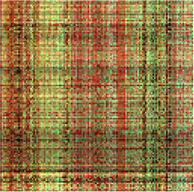	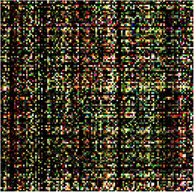	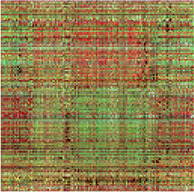	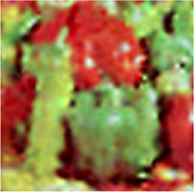	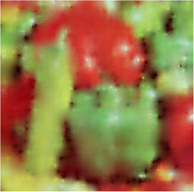
[6.2,0.01,0.97]	[10,0.3,0.6]	[12.2,0.45,0.5]	[4,0.03,1.2]	[13.1,0.5,0.4]	[19,0.85,0.22]	[**19.52,0.86,0.21**]

**TABLE 4:** Comparison of the performance of the algorithms for the reconstruction of images with 99% missing ratio. PSNR, SSIM and normalized approximation error corresponding to each image have been written in brackets.

Missed image	HaLRTC	TT-WOPT	TR-ALS	TR-LRF	MDT	Proposed
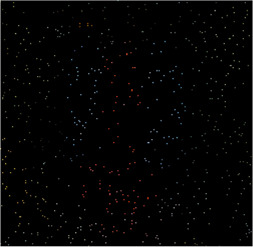	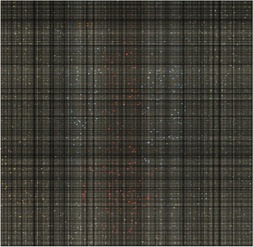	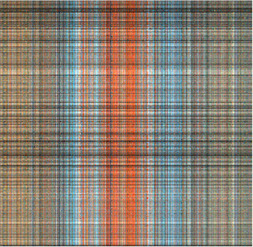		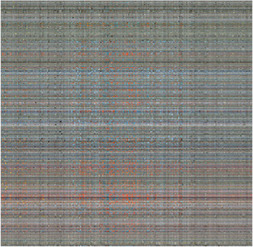	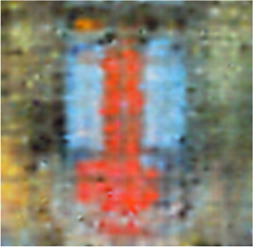	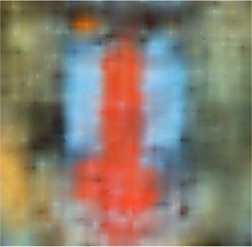
[5.44,0.002,0.99]	[9.25,0.1,0.64]	[13.3,0.2,0.4]	Fail	[13.45,0.1,0.39]	[18.04,0.54,0.23]	[**18.34,0.55,0.22**]
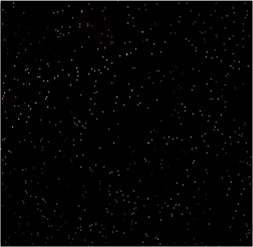	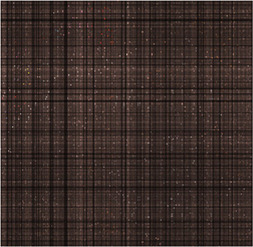	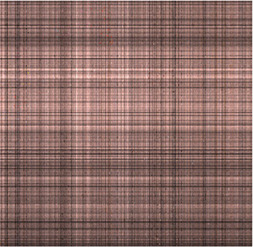		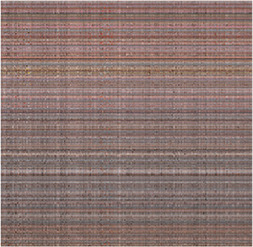	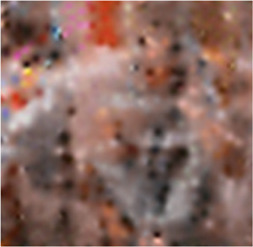	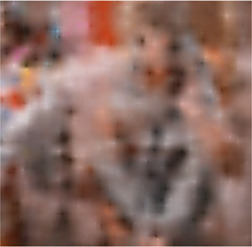
[6.48,0.002,0.99]	[9.9,0.2,0.67]	[13.5,0.3,0.44]	Fail	[14.75,0.3,0.4]	[18.41,0.67,0.25]	[**18.62,0.68,0.24**]
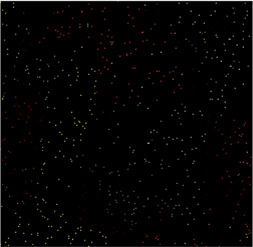	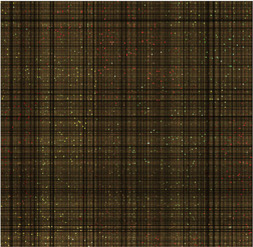	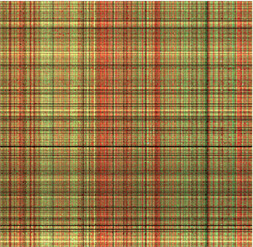		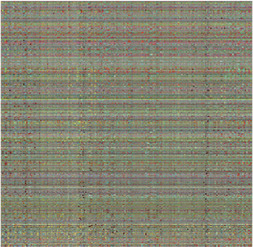	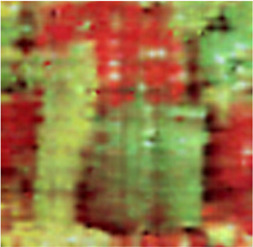	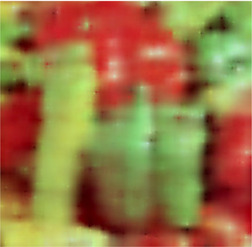
[5.96,0.002,0.99]	[8.8,0.23,0.71]	[11.9,0.46,0.5]	Fail	[11.84,0.23,0.5]	[17.3,0.81,0.27]	[**18.15,0.83,0.24**]

In the next simulation, the algorithms have been compared for the completion of structurally missing images. The images with blocks and slices missing elements were completed by the algorithms and the results have been presented in Table 5. The image size of this simulation was 128×128×3 and the algorithms parameters were similar to Table 1 for 90% missing ratio. The completed images along with the PSNR’s, SSIM’s and normalized approximation errors, show the superiority of the proposed approach in comparison to the other completion methods.

**TABLE 5:** Comparison of the performance of the algorithms for the reconstruction of images with structural missing elements. PSNR, SSIM and normalized approximation error corresponding to each image have been written in brackets.

Missed image	HaLRTC	TT-WOPT	TR-ALS	TR-LRF	MDT	Proposed
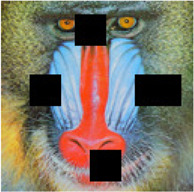	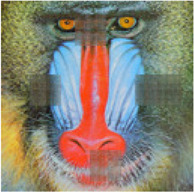	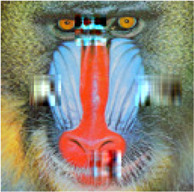	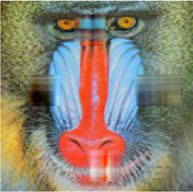	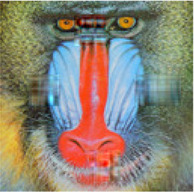	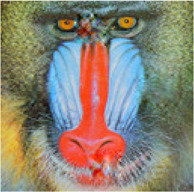	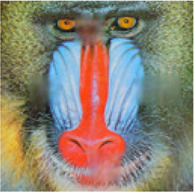
[15.3,0.84,0.3]	[25.3,0.9,0.1]	[19,0.9,0.2]	[24.5,0.9,0.1]	[25.33,0.9,0.1]	[25.8,0.94,0.09]	[**28.31,0.95,0.07**]
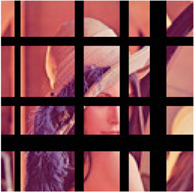	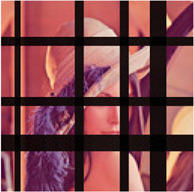	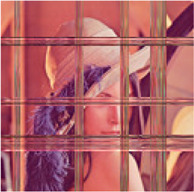		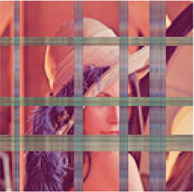	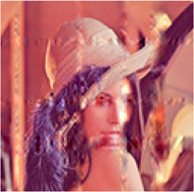	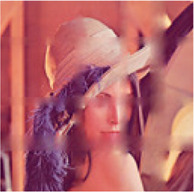
[9.5,0.5,0.6]	[10.5,0.6,0.5]	[18.2,0.84,0.2]	Fail	[17.6,0.7,0.2]	[21.96,0.93,0.14]	[**24.28,0.95,0.11**]

In the next simulation, the effectiveness of the multistage strategy has been investigated. For this purpose, one stage algorithms with P=8 and P=4 have been compared with a two stage algorithm with $P_{1}=8$ and $P_{2}=4$ for the completion of an image with 99% missing ratio. The window size was set to t=[2,2] and the initial rank vector for the second stage was set to [10,10,…,10]. The results have been illustrated in Table 6. As the results show, the quality of the reconstructed image resulted from the two stage algorithm is higher than the images resulted from single stage algorithms. This is because the first stage of the algorithm can provide a good initialization for the second stage, which can result in a better completion performance comparing to single stage algorithms.

**TABLE 6:** Investigation of the effectiveness of the multistage strategy. Single stage algorithms with P=8 and P=4 have been compared with a multistage algorithm with $P_{1}=8$ and $P_{2}=4$. PSNR, SSIM and normalized approximation error corresponding to each figure have been written in brackets.

Missed image	One stage algorithm (P=8)	One stage algorithm (P=4)	Two stage algorithm ($P_{1}=8$, $P_{2}=4$)
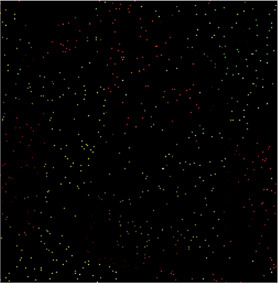	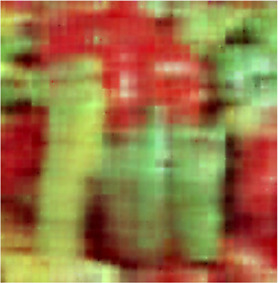	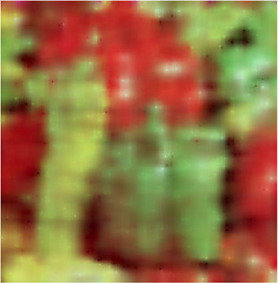	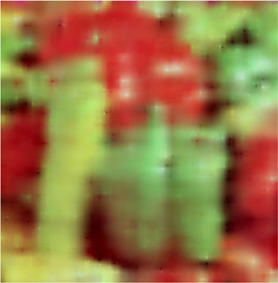
[5.96,0.002,0.99]	[17.81,0.82,0.25]	[17.66,0.81,0.27]	[**18.15,0.83,0.24**]

Finally, for investigating the effect of noise on the performance of the proposed algorithm, several incomplete noisy images have been completed by the proposed and the MDT approaches. The size of the images in this simulation was 128×128×3. The missing ratio of the images was 90% and the remaining pixels have been contaminated by noise with normal distribution and standard deviation *σ*. The two methods have been compared for different *σ*’s. The parameters of the two algorithms were similar to Table 1 for 90% missing ratio. The ranks of the second stage of the proposed algorithm have been set equal to 10 for σ=0.1 and 3−6 for other *σ*’s. For the MDT approach, noise levels have been selected as $10^{-2}$, $4\times 10^{-2}$, $10^{-1}$, $1.6\times 10^{-1}$, $2.5\times 10^{-1}$ and $3.6\times 10^{-1}$ for σ=0.1,0.2,0.3,0.4,0.5 and 0.6, respectively. For each *σ*, seven incomplete noisy images have been completed by each algorithm and the resulting PSNR’s have been averaged and the standard deviation has been computed. Note that in noisy cases, the line 12 of the Algorithm 1 moves to after line 13 and the output of the line 13 will be the output of each stage (i.e., before replacing the observed elements). The results have been presented in Table 7. As the results show, the qualities of the both approaches decrease by increasing *σ*. However, even in these noisy cases, the quality of the proposed approach is higher than MDT.

**TABLE 7:** Investigation of the effect of noise on the performance of the proposed and MDT algorithms. Images have 90% missing ratio and *σ* is the standard deviation of noise. The presented results are the averaged PSNR’s over seven incomplete noisy images and their standard deviations. It is clear that even in noisy cases, the proposed approach outperforms MDT.

*σ*	0.1	0.2	0.3	0.4	0.5	0.6
Proposed algorithm	21.16 ± 0.61	19.80 ± 0.53	18.55 ± 0.61	18.02 ± 0.74	17.45 ± 0.76	16.54 ± 0.69
MDT	20.78 ± 0.84	19.39 ± 0.53	18.1 ± 0.67	17.69 ± 0.74	17.31 ± 0.95	16 ± 0.62

## 7 Discussion

A new approach for image completion in the embedded space by block Hankelization and by exploiting TR decomposition has been proposed and extensively tested. In this approach, the incomplete image has been transformed into a higher order 7-th order tensor using block Hankelizaion and then a TR decomposition with rank incremental strategy and smoothness have been applied for completion. Moreover, a multistage strategy, which has been previously applied by us for time series completion, has been used for increasing the quality of the final completed image. Simulation results and comparisons with the state of the art algorithms indicated the advantage of the proposed algorithm.

## Data Availability

The original contributions presented in the study are included in the article/Supplementary Material, further inquiries can be directed to the corresponding author.
